# Estimating the Intracluster Correlation Coefficient for the Clinical Sign “Trachomatous Inflammation—Follicular” in Population-Based Trachoma Prevalence Surveys: Results From a Meta-Regression Analysis of 261 Standardized Preintervention Surveys Carried Out in Ethiopia, Mozambique, and Nigeria

**DOI:** 10.1093/aje/kwz196

**Published:** 2019-09-11

**Authors:** Colin K Macleod, Robin L Bailey, Michael Dejene, Oumer Shafi, Biruck Kebede, Nebiyu Negussu, Caleb Mpyet, Nicholas Olobio, Joel Alada, Mariamo Abdala, Rebecca Willis, Richard Hayes, Anthony W Solomon

**Affiliations:** 1 Clinical Research Department, London School of Hygiene & Tropical Medicine, London, United Kingdom; 2 Michael Dejene Public Health Consultancy Services, Addis Ababa, Ethiopia; 3 Federal Ministry of Health, Addis Ababa, Ethiopia; 4 Department of Ophthalmology, Queen Mamohato Memorial Hospital, Maseru, Lesotho; 5 Sightsavers, Kaduna, Nigeria; 6 Kilimanjaro Centre for Community Ophthalmology International, Division of Ophthalmology, Groote Schuur Hospital, Cape Town, South Africa; 7 National Trachoma Control Program, Department of Public Health, Federal Ministry of Health, Abuja, Nigeria; 9 Ophthalmology Department, Ministry of Health, Maputo, Mozambique; 10 Task Force for Global Health, Decatur, Georgia; 11 MRC Tropical Epidemiology Group, Department of Infectious Disease Epidemiology, London School of Hygiene & Tropical Medicine, London, United Kingdom; 12 Department of Control of Neglected Tropical Diseases, World Health Organization, Geneva, Switzerland

**Keywords:** clustering, intracluster correlation coefficient, prevalence, surveys, trachoma, trachomatous inflammation—follicular

## Abstract

Sample sizes in cluster surveys must be greater than those in surveys using simple random sampling in order to obtain similarly precise prevalence estimates, because results from subjects examined in the same cluster cannot be assumed to be independent. Therefore, a crucial aspect of cluster sampling is estimation of the intracluster correlation coefficient (ρ): the degree of relatedness of outcomes in a given cluster, defined as the proportion of total variance accounted for by between-cluster variation. In infectious disease epidemiology, this coefficient is related to transmission patterns and the natural history of infection; its value also depends on particulars of survey design. Estimation of ρ is often difficult due to the lack of comparable survey data with which to calculate summary estimates. Here we use a parametric bootstrap model to estimate ρ for the ocular clinical sign “trachomatous inflammation—follicular” (TF) among children aged 1–9 years within population-based trachoma prevalence surveys. We present results from a meta-regression analysis of data from 261 such surveys completed using standardized methods in Ethiopia, Mozambique, and Nigeria in 2012–2015. Consistent with the underlying theory, we found that ρ increased with increasing overall TF prevalence and smaller numbers of children examined per cluster. Estimates of ρ for TF were independently higher in Ethiopia than in the other countries.

## Abbreviations

CI: confidence interval
GPS: Global Positioning System
GTMP: Global Trachoma Mapping Project
MDA: mass drug administration
PSU: primary sampling unit
TF: trachomatous inflammation—follicular

## 

Trachoma is a blinding disease caused by infection with the bacterium *Chlamydia trachomatis.* Ocular infection is mostly found in young children, with repeated infections leading to chronic keratoconjunctivitis (1, 2). Over a period of years, immunologically mediated scarring of the eyelid occurs, causing permanent changes in eyelid morphology and misdirection of the eyelashes so that they abrade the front surface of the eye, leading to permanent opacification of the cornea. Standardized clinical signs of trachoma, defined according to the World Health Organization’s simplified trachoma grading system (3), are used to provide reproducibility in surveys. In this system, “trachomatous inflammation—follicular” (TF) is defined as the presence of 5 or more follicles, each greater than or equal to 0.5 mm in diameter, in the central part of the tarsal conjunctiva of the upper eyelid. Estimates of the prevalence of TF in children aged 1–9 years are used to guide intervention planning and, in particular, to decide where and for how long to implement annual mass distribution of azithromycin, the antibiotic used to treat trachoma.

From 2012 to 2015, standardized baseline prevalence surveys took place throughout Ethiopia, Nigeria, and Mozambique as part of the Global Trachoma Mapping Project (GTMP), with the aim of identifying districts that needed interventions in a push toward global trachoma elimination. These surveys provided data that have been made available to further analysis, to augment existing knowledge of trachoma epidemiology, and to refine future survey protocols for greatest efficiency and accuracy.

Trachoma is found in isolated, socioeconomically deprived rural areas. Population-based prevalence surveys are the gold standard for evaluating its prevalence (4). Although ideally one would select individuals to be examined at random from the target population, so that all residents were equally likely to be selected (simple random sampling), survey costs can be reduced by instead selecting clusters of individuals within geographical locales (cluster sampling). This increases fieldwork efficiency at the expense of the statistical independence of each result. To compensate for the relatedness of individuals within a given cluster and the resulting increased variance in estimates produced as a result of the cluster-sampled design, sample sizes must be increased. The parameter used to describe the correlation of results from individuals within a given cluster is known as the intracluster correlation coefficient (ρ), defined as the proportion of total variance accounted for by between-cluster variation. In infectious disease epidemiology, this coefficient is associated with transmission patterns and the natural history of infection and may depend on the particulars of survey design. An accurate estimate of ρ is needed to design future surveys and, in particular, to determine an appropriate sample size.

In this paper, we use parametric bootstrapping to estimate ρ with 95% confidence intervals for each of 261 trachoma prevalence surveys from Ethiopia, Nigeria, and Mozambique. These estimates are then used to conduct a meta-regression analysis with survey-level covariates to explore variation across surveys and to investigate the influence of key factors on ρ.

## METHODS

### Sampling design

All surveys were carried out using standardized methodology as part of the GTMP (5). A planned sample size of 1,019 children aged 1–9 years was used to estimate an expected TF prevalence of 10% with a precision of ±3% at the 95% confidence level, using a design effect (the ratio of the clustered sampling variance to simple random sampling variance) of 2.65, the latter being derived from surveys carried out prior to the GTMP.

At the first stage of sampling, primary sampling units (PSUs) were identified in each district. The number of households sampled per PSU (*h*) was set as that which a single survey team could anticipate being able to sample in 1 working day: 25 in Nigeria, 30 in Ethiopia, and 32 in Mozambique. The number of PSUs in each survey was then dependent on the mean number of children aged 1–9 years that were expected to be found in each household, ${n}_H$, with the number of PSUs equal to 1,019/(*h* × ${n}_H$). This meant that 24–26 PSUs were planned per survey. Typically, existing census data were used to define the sampling frame for PSUs, the resolution being limited by the population size of the lowest administrative census units in the country. PSUs were villages, groups of villages, or other administrative areas. PSUs were sampled with a probability-proportional-to-size methodology, giving more weight to larger (more populous) PSUs. This provided self-weighting of samples so that, despite the clustered design, each individual in the evaluation unit had (as far as was practically possible) an equal likelihood of being sampled.

At the second stage of sampling, within the PSU, compact segment sampling (Ethiopia and Mozambique) or random-walk sampling (Nigeria) was used to select households for inclusion. In Ethiopia and Mozambique, each PSU was divided into segments of 30 and 32 contiguous households, respectively, so that each household in the PSU belonged to a segment. One segment was then chosen at random by drawing lots. All individuals resident in the households of the chosen segment were visited by the survey team. In Nigeria, using random-walk sampling, a starting point in the center of the PSU was agreed upon and a pen was spun on the ground at that point to identify, in quasirandom fashion, a heading for the survey team to transect. A total of 25 households in that direction were enrolled.

In sampled households, all residents aged ≥1 year were eligible for inclusion, and all consenting individuals were examined for signs of trachoma using the World Health Organization’s simplified trachoma grading system (3). For children under age 18 years, consent was obtained from the parent or guardian, and the children themselves gave assent where possible. Data were collected electronically on Android smartphones (Google, Inc., Mountain View, California) (5).

### Ethical clearance

The overall GTMP protocol was approved by the ethics committee of the London School of Hygiene & Tropical Medicine. In Ethiopia, the protocol was approved by the ethics committee of each participating regional state. In Mozambique, the protocol was approved by the National Committee on Bio-Ethics and the Provincial Directorate of Health in each province. In Nigeria, the protocol was approved by the National Health Research Ethics Committee. The secondary analyses of anonymized data that underlie this paper were considered by the Ethics Review Committee of the World Health Organization to be exempt from full formal review.

### Ethiopia

Between December 2012 and May 2015, a total of 168 standardized surveys were carried out in 7 regions—Afar; Benishangul-Gumuz; Gambella; Oromia; Somali; Southern Nations, Nationalities, and Peoples’ Region; and Tigray. Survey environments ranged from deserts in the Somali region to the highlands of Tigray and tropical rainforests in Gambella. Results of these surveys have been published elsewhere (6–10).

### Nigeria

Between February 2013 and February 2014, 121 standardized surveys were carried out in Katsina, Kano, Bauchi, and Kaduna states. The results of these surveys have been published elsewhere (11–14).

### Mozambique

Between December 2011 and June 2015, 91 standardized surveys were carried out in Cabo Delgado, Gaza, Inhambane, Manica, Maputo, Nampula, Niassa, Sofala, Tete, and Zambezia provinces. The results of these surveys have been published elsewhere (15).

### 

**Estimating** $\boldsymbol{rho}$

The standard equation for the variance of a proportion achieved through simple random sampling (SRS) of *N* individuals is given by ${\mathrm{Var}}_{\mathrm{SRS}}(p)=\frac{pi \big(1-pi \big)}{N}$, where *p* is the sample proportion of the outcome, π is the true proportion of the outcome in the whole population, and *N* is the total number of individuals examined. In cluster sampling, the increased variance arising from the clustered design is represented by the design effect (DE),

(1) \begin{equation*}\mathrm{DE}=[1+(m-1)rho ],\end{equation*}

so that

$$\begin{equation*}{\mathrm{Var}}_{\mathrm{Cluster}}(\,\overline{p})=\frac{pi (1-pi)}{nm}[1+(m-1)rho ],\end{equation*}$$

where

$$\begin{equation*}\overline{p}=\frac{1}{n}{\sum}_{i=1}^n{p}_i.\end{equation*}$$

Here *n* is the number of clusters in the survey and *m* is the average number of individuals examined per cluster. Hence, *nm* = *N*, the total number of individuals examined.

Therefore,

$$\begin{equation*}\hat{rho}=\frac{\left(\frac{{\mathrm{Var}}_{\mathrm{Cluster}}\big(\bar{p}\big)}{{\mathrm{Var}}_{\mathrm{SRS}}\big(\bar{p}\big)}\right)-1}{m-1},\end{equation*}$$

where ${\mathrm{Var}}_{\mathrm{SRS}}(p)$ is approximated as ${\mathrm{Var}}_{\mathrm{SRS}}\big(\overline{p}\big)$. We therefore need to estimate ${\mathrm{Var}}_{\mathrm{Cluster}}\big(\overline{p}\big)$ to calculate $\hat{rho}$ for a given survey.

### Estimating the between-cluster variance in *p*

We used parametric resampling with replacement (parametric bootstrapping) to estimate ${\mathrm{Var}}_{\mathrm{Cluster}}\big(\overline{p}\big)$. Parametric resampling makes no assumptions about the underlying distribution of the data (16), but the resampling process should mirror, where possible, the sampling strategy that gave rise to the data (17, 18).

The data can be represented as a vector of *N* independent observations, ${\mathbf{y}}_{\mathrm{obs}}$. We wish to estimate the variance of the parameter $p\big({\mathbf{y}}_{\mathrm{obs}}\big)$ by replicating the highest-level sampling strategy used in the surveys. In this secondary analysis of deidentified data sets, the underlying populations of selected clusters were not known, so equal weighting (rather than weighting proportional to size) was used.

For each $\hat{rho}$ estimate, the following algorithm was used:

1. Determine the number of unique clusters in the survey, *n*, and sample *n* clusters randomly with replacement. All children aged 1–9 years examined in these clusters comprise the bootstrap data set ${Y}^{\ast }$. Let *i* = 1, 2, … *n*.
2. Calculate the cluster-level TF proportion of ${p_i}^{\ast }$ as the sum of all cluster TF cases divided by the number of children examined in the cluster.
3. Calculate the bootstrap prevalence estimate $\overline{p}\big({Y}^{\ast}\big)$ as $\frac{1}{n}{\sum}_1^n{p_i}^{\ast }$ (the mean of all *n* cluster-level proportions).
4. Repeat steps 1–4 a total of 4,096 times to generate an estimate of the bootstrap distribution of Y^*^.
5. ${\mathrm{Var}}_{\mathrm{Cluster}}\big(\overline{p}\big)$ is estimated as the variance of this bootstrap distribution.
6. For each survey, $\hat{rho}$ is then estimated as $\frac{\left(\frac{\mathrm{Var}\ {\big(\bar{p}\big)}_{\mathrm{Cluster}}}{\frac{\bar{p}\big(1-\bar{p}\big)}{N}}\right)-\,1\ }{\big(m-1\big)}$.
7. The variance of $\hat{rho}$ is estimated by replicating steps 1–6 a total of 4,096 times.

In our analysis, bootstrap distributions approximated normal distributions, so 95% confidence intervals were calculated as the 2.5th and 97.5th percentiles of all ordered estimates for a given survey. The overall estimate for each survey was the mean value of these estimates. Bootstrap estimates were resampled 4,096 (2^12^) times to obtain appropriate precision. A total of 4,096^2^ replications were carried out for each $\hat{rho}$ estimate. Estimation was carried out in RStudio (RStudio, Inc., Boston, Massachusetts).

### Meta-analysis

Next, we conducted a meta-analysis to obtain pooled estimates of ρ across surveys. Pooled estimates were derived using a random-effects model, with survey weights obtained from the intrasurvey variance of each estimate (19, 20). Natural log-transformed estimates were used to limit the effects of heteroscedasticity. Heterogeneity across survey estimates was investigated using the *Q* statistic, subgroup analysis, and meta-regression analyses (21). Random-effects meta-regression models were fitted to estimates using the “metareg” command in STATA 14 (StataCorp LLC, College Station, Texas). The standard error of each estimate was calculated as the difference between the 97.5th and 2.5th centile estimates divided by 3.92, assuming a normal distribution of bootstrap estimates. Estimates of ρ are reported on the original scale by exponentiating the pooled estimates from the model. Design effect estimates at given covariate values were estimated from pooled ρ estimates as $1+\big(m-1\big)rho$, with *m* set as 30 children per cluster. Forest plots were produced in STATA 14.

### Analysis plan

We excluded surveys in which the TF prevalence estimate was less than 2%, in the belief that below this level the data would be too sparse to reliably estimate ρ. We used univariate and multivariable meta-regression techniques to investigate possible sources of heterogeneity between estimates, using the following covariates: TF prevalence, country, mean distance between clusters, mean number of children examined per household, and mean number of children examined per cluster. Covariates were defined using data collected at the time of the survey. For each survey, the average distance between clusters was estimated as the difference between the respective Global Positioning System (GPS) coordinates of each cluster and the centroid GPS coordinates over all clusters, with estimates adjusted for latitude to convert decimal degrees to kilometers. We included this covariate to test the hypothesis that survey areas that covered larger distances were more likely to show a greater variance in TF estimates. We then conducted secondary analyses using ρ estimates stratified by associated covariates.

At the time of data collection, recorders entering data into smartphones were required to submit a unique identity code. This allowed the total number of data recorders to be defined for each survey. Because recorders were paired with graders performing clinical trachoma grading, we included this variable to investigate trachoma grader precision or consistency between graders in a given survey.

## RESULTS

A total of 380 surveys from Ethiopia, Nigeria, and Mozambique were made available by the respective health ministries. We excluded 111 surveys because their TF prevalence was below the 2% threshold. We further excluded another 8 surveys because they had an estimated ρ value less than 0.0. Thus, 261 surveys were included in the analysis: 162 from Ethiopia, 44 from Mozambique, and 55 from Nigeria (see Web Table 1, available at https://academic.oup.com/aje). All included surveys used a 2-stage cluster sample survey design. All survey data were baseline trachoma prevalence estimates, with none of the surveyed populations having received previous mass azithromycin administration or other specific interventions deployed to reduce active trachoma prevalence by national elimination programs.

The TF prevalence in children aged 1–9 years was reported in the surveys as the mean of all cluster-level proportions. The median TF prevalence in children aged 1–9 years over all surveys was 16.5% (interquartile range, 4.5–27.5; range, 2.0–50.6). The breakdown of survey-level prevalence by country is shown in Web Table 2.

### Number of children examined per cluster

The mean number of children aged 1–9 years examined per cluster was 36.6 in Ethiopia, 39.6 in Mozambique, and 69.1 in Nigeria. Full details of the breakdown of cluster sizes by country are shown in Web Table 3.

### Number of children examined per household

The number of children examined per household was considered in the analysis because larger households may have an effect on trachoma transmission either through proximity and interpersonal interaction as a direct risk factor or through common exposures, such as the effect of poor community-level access to sanitation (22). The mean number of children aged 1–9 years examined per household was 2.0 in Ethiopia, 2.0 in Mozambique, and 3.1 in Nigeria (Web Table 4).

### Initial meta-analysis

**Figure 1:**
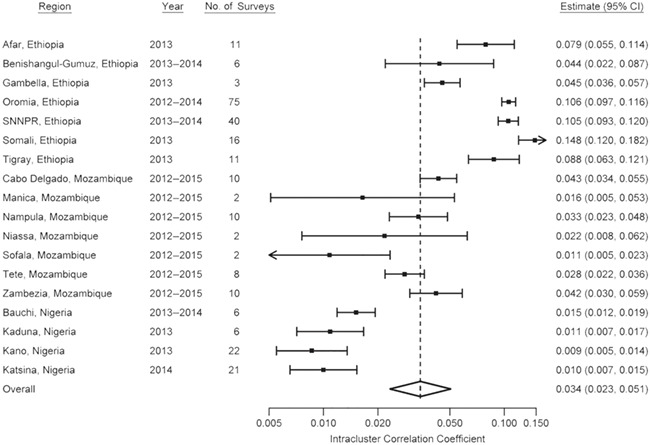
Region-level summary forest plot of bootstrap estimates of the intracluster correlation coefficient for 261 standardized population-based trachoma prevalence surveys carried out in Ethiopia, Mozambique, and Nigeria, Global Trachoma Mapping Project, 2012–2015. Bars, 95% confidence intervals (CIs). SNNPR, Southern Nations, Nationalities, and People’s Region.

The meta-analysis included 261 estimates of ρ for the clinical sign TF in children aged 1–9 years. The region-level estimates across all surveys are shown in Figure 1. Estimates ranged from 0.0002 (95% confidence interval (CI): 0.0000, 0.0008) in a survey in Kano State, Nigeria, to 0.368 (95% CI: 0.348, 0.388) in a survey in the Southern Nations, Nationalities, and People’s Region of Ethiopia. The overall pooled estimate for all surveys was 0.051 (95% CI: 0.047, 0.056), although there was a great deal of heterogeneity in ρ between surveys (heterogeneity χ^2^ = 120,000; *P* < 0.0001).

The largest and least precise estimates were generally from Ethiopia. The pooled ρ estimate was 0.100 (95% CI: 0.093, 0.108) in Ethiopia, 0.033 (95% CI: 0.027, 0.039) in Mozambique, and 0.009 (95% CI: 0.007, 0.012) in Nigeria. When stratified by TF prevalence (within groupings used for making intervention decisions according to World Health Organization recommendations (23)), the pooled estimates were 0.015 (95% CI: 0.012, 0.020), 0.033 (95% CI: 0.026, 0.042), 0.081 (95% CI: 0.071, 0.092), and 0.111 (95% CI: 0.101, 0.124) for TF prevalences of <5.0%, 5.0%–9.9%, 10.0%–29.9%, and ≥30.0%, respectively.

The heterogeneity across country-specific estimates remained even after stratification by TF prevalence. The respective pooled ρ estimates for TF prevalences of <5.0%, 5.0%–9.9%, 10.0%–29.9%, and ≥30.0% were 0.042 (95% CI: 0.029, 0.062), 0.103 (95% CI: 0.084, 0.127), 0.105 (95% CI: 0.093, 0.119), and 0.114 (95% CI: 0.104, 0.126) in Ethiopia and 0.007 (95% CI: 0.005, 0.009), 0.008 (95% CI: 0.005, 0.014), 0.025 (95% CI: 0.015, 0.040), and 0.022 (95% CI: 0.020, 0.024) in Nigeria. In Mozambique, the pooled estimates for TF prevalences of 5.0%, 5.0%–9.9%, and 10.0%–29.9% were 0.022 (95% CI: 0.015, 0.032), 0.033 (95% CI: 0.024, 0.044), and 0.055 (95% CI: 0.044, 0.070), respectively; no survey in Mozambique estimated a TF prevalence of ≥30.0%.

In the univariate meta-regression analyses, a large proportion of variability across all 261 ρ estimates could be explained by country, TF prevalence, mean distance between clusters, number of recorders used in the survey, number of children examined per household, and number of children examined per cluster (Table 1). A larger ρ estimate was associated with a higher TF prevalence, a larger distance between clusters, a larger number of recorders used in the survey, a smaller number of children examined per household, and a smaller number of children examined per cluster. Estimates were generally highest in Ethiopia and lowest in Nigeria.

**Table 1:** Results From Meta-Regression of the Intracluster Correlation Coefficient for the Clinical Sign “Trachomatous Inflammation—Follicular” Among Children Aged 1–9 Years in 261 Population-Based Trachoma Prevalence Surveys, Ethiopia, Mozambique, and Nigeria, 2012–2015

**Covariate**	**No. of** **Surveys**	**Pooled ρ** **Estimate** ^**a**^	**95% CI**	**% of Variance Explained** ^**b**^	**β** ^**c**^ ^**,**^ ^**d**^	**95% CI**
Country				58.2		
Ethiopia	162	0.100	0.077, 0.128		1.000	Referent
Mozambique	44	0.032	0.023, 0.045		0.471	0.348, 0.638
Nigeria	55	0.010	0.008, 0.012		0.789	0.273, 2.281
No. of children examined per cluster				44.7		
15–29	36	0.097	0.071, 0.132		1.000	Referent
30–49	172	0.071	0.050, 0.100		0.882	0.658, 1.181
50–79	34	0.016	0.010, 0.024		0.518	0.332, 0.809
≥80	19	0.005	0.003, 0.008		0.212	0.096, 0.470
TF^e^ prevalence, %				36.2		
<5.0	56	0.015	0.011, 0.020		1.000	Referent
5.0–9.9	56	0.033	0.023, 0.048		1.638	1.230, 2.181
10.0–29.9	87	0.080	0.057, 0.114		2.392	1.816, 3.150
≥30.0	62	0.111	0.077, 0.162		2.493	1.810, 3.432
No. of recorders^f^				48.7		
<5	53	0.009	0.007, 0.012		1.000	Referent
5–9	43	0.057	0.039, 0.083		2.335	0.834, 6.534
10–19	149	0.082	0.061, 0.109		2.740	0.945, 7.944
≥20	16	0.106	0.063, 0.178		2.727	0.891, 8.343
No. of children examined per household				30.1		
1.0–1.9	105	0.081	0.066, 0.099		1.000	Referent
2.0–2.9	130	0.051	0.038, 0.067		0.786	0.627, 0.986
3.0–3.9	19	0.006	0.003, 0.010		0.721	0.387, 1.339
≥4.0	7	0.010	0.004, 0.023		1.710	0.677, 4.316
Quartile of distance between clusters^g^, km				13.8		
1	65	0.023	0.017, 0.030		1.000	Referent
2	66	0.051	0.034, 0.076		0.974	0.734, 1.289
3	65	0.084	0.056, 0.127		1.059	0.785, 1.430
4	65	0.065	0.043, 0.098		0.963	0.700, 1.324

Abbreviations: CI, confidence interval; GPS, Global Positioning System; ICC, intracluster correlation coefficient; TF, trachomatous inflammation—follicular.

^a^Pooled estimate of the ICC.

^b^Proportion of the variability between survey ICC estimates explained by each covariate on the natural logarithmic scale (*P* < 0.0001 for all variables).

^c^Exponentiated meta-regression coefficient.

^d^Full meta-regression model adjusting for country, number of children examined per cluster, and prevalence of TF in children aged 1–9 years (*P* < 0.0001). 69.2% of the variance in the ICC was explained by the full model.

^e^TF in children aged 1–9 years, the primary clinical sign associated with ocular *Chlamydia trachomatis* infection used to guide intervention programs under current World Health Organization guidelines (23).

^f^Estimated as the number of unique recorder identification codes used in the survey.

^g^Estimated as the square root of the variance of the distance of survey clusters from the geometric center of the GPS coordinates of all survey clusters, converted to kilometers and accounting for latitude.

The multivariable meta-regression analyses aimed to explain the heterogeneity between surveys, accounting for survey-level differences in associated variables. The country covariate was included in the model a priori. When controlling for all variables in the model, only country, TF prevalence category, and cluster size were associated with ρ (*P* < 0.001), explaining 69.8% of the variability. Ethiopia was independently associated with higher estimates (β = 2.39 (95% CI: 1.85, 3.07); *P* < 0.001), with no meaningful difference between Mozambique and Nigeria (*P* = 0.934). The “number of children examined per household” covariate was not included in the final model because of collinearity with the “number of children examined per cluster” covariate. The “number of recorders used per survey” covariate was not included because of collinearity with the country covariate (the Ethiopia and Nigeria surveys were perfectly collinear with number of recorders <5 and number of recorders ≥20, respectively). The final multivariable model accounted for 69.2% of the variance in estimates (Table 1).

## DISCUSSION

In general, the intracluster correlation coefficient or the design effect is poorly represented in the public health literature. Individual survey clustering estimates exist (24–27), but we have found only 1 other paper that covered clustering estimates derived from surveys carried out in multiple countries (28). We believe this to be the first time that estimates of ρ from standardized infectious disease surveys conducted internationally have been published together.

Surveys of a particular infectious disease are not always standardized, and as a result it has not previously been possible to amass large numbers of comparable pooled estimates of ρ in a single analysis. We have therefore had an opportunity to augment existing knowledge in a way that was not possible for trachoma prior to the implementation of the GTMP. We found marked heterogeneity in survey ρ estimates, and we explored possible sources of that heterogeneity which may be of use in planning future work.

In 1996, the World Health Organization targeted trachoma for elimination as a public health problem by the year 2020 (29). This was defined, in part, as an estimated TF prevalence in children aged 1–9 years of less than 5% in each formerly endemic district. An important aspect of validating that this goal has been reached is confidence in the method by which prevalence has been measured. Given the marked effect that the ρ estimate has on sample-size planning, it is crucial to have accurate estimates of its value. We have shown that ρ decreases sharply at low TF prevalences, and so with the same absolute precision, accurate estimates of TF can be made using smaller sample sizes as the anticipated elimination endpoint approaches. The converse of this statement is that for a given sample size, with increasing TF prevalence, the precision of a given estimate decreases. In trachoma elimination, the crucial TF thresholds are 5%, 10% and 30%: Where TF prevalence is less than 5.0%, azithromycin mass drug administration (MDA) is not indicated; where it is 5.0%–9.9%, a single round of MDA is recommended before resurvey; where it is 10.0%–29.9%, 3 annual rounds of MDA are recommended before resurvey; and where it is 30.0% or more, 5 annual rounds of MDA are recommended before resurvey. The required performance of a survey methodology for providing estimates around these thresholds depends on the implications of erroneous categorization to the population involved. Incorrect categorization may have significant implications around the 10% threshold, for example, where the cost difference between implementing 1 and 3 years of MDA and the political effect of delaying repeat surveys may each be substantial.

On univariate analysis, there was a suggestion that using fewer data recorders in a given survey was associated with greater concordance of cluster-level TF estimates, and so decreased ρ. However, this variable was not retained in the full multivariable model with the country variable included. It is possible that there was not enough variability in recorder numbers within countries to obtain accurate estimates independent of the overall country variable. From the data, it can be inferred that local logisticians used different field team deployment strategies for completing large numbers of surveys in a given area. One strategy was to use a single data recorder (and, generally, a single accompanying trachoma grader) for a whole survey, so that the individual worked in all clusters in the evaluation unit: If 26 clusters were required, the survey would take 26 team-days of fieldwork for that recorder and his or her trachoma grader. This strategy was used in the majority of surveys in Nigeria and Mozambique. The strategy at the other extreme would be to send 26 data recorders (and their accompanying graders) to 1 cluster each, so that the survey could in theory be completed in a single calendar day (still incorporating 26 team-days of fieldwork). The strategy used in Ethiopia was closer to this model. Intuitively, the trade-off between these strategies is the trade-off between accuracy and precision. One team might be inaccurate, but if so it might be reliably inaccurate and therefore give precision to estimates (and concordance between results). The mean of the cluster-level TF proportions might not necessarily be close to the true population estimate. On the other hand, multiple teams contributing to a single survey could all be inaccurate, but the mean of the cluster-level proportions derived from many hands might (or might not) be closer to the true population-level estimate of disease prevalence. Although the number of recorders was not included in the final model in this analysis, it is possible that this could be considered as a variable in future analyses.

A limitation of this analysis in guiding future surveys is that in the populations surveyed here, for districts in which the TF prevalence was at least 5%, interventions against active trachoma will have been deployed before impact surveys are conducted, and the degree to which the preintervention epidemiology of trachoma is representative of its postintervention epidemiology is unclear, as the varying interventions may have varying impacts on the epidemiology of the underlying disease. Equally uncertain is whether these data will be externally applicable in countries yet to complete baseline trachoma mapping of suspected trachoma-endemic districts.

Overall, we found large variation in ρ estimates between surveys, and so we recommend that ρ estimates used for planning future surveys be conservative. In other words, overestimating the assumed value of ρ would be epidemiologically prudent.

It is hoped that these data can be used to guide future trachoma programs to aid elimination efforts. However, for programmatic use, the design effect is a more commonly cited parameter than ρ, as it is more intuitively useful for program managers, being the factor by which a simple random-sampling sample size should be multiplied to provide equivalent precision in a cluster random sample. Using equation 1, our analyses suggest that when carrying out surveys with more than 30 children examined per cluster, a design effect greater than 2.6 should be used when a TF prevalence close to 5% is expected, a design effect greater than 3.6 should be used when a TF prevalence close to 10% is expected, and a design effect greater than 5.0 should be used when a TF prevalence close to 30% is expected.
